# Review of Membranes for Helium Separation and Purification

**DOI:** 10.3390/membranes7010009

**Published:** 2017-02-17

**Authors:** Colin A. Scholes, Ujjal K. Ghosh

**Affiliations:** 1 Department of Chemical & Biomolecular Engineering, The University of Melbourne, Melbourne, VIC 3010, Australia; 2 Department of Chemical Engineering, College of Engineering, Qatar University, Doha 2713, Qatar; ughosh@qu.edu.qa

**Keywords:** helium, membranes, polymeric, selectivity, process

## Abstract

Membrane gas separation has potential for the recovery and purification of helium, because the majority of membranes have selectivity for helium. This review reports on the current state of the research and patent literature for membranes undertaking helium separation. This includes direct recovery from natural gas, as an ancillary stage in natural gas processing, as well as niche applications where helium recycling has potential. A review of the available polymeric and inorganic membranes for helium separation is provided. Commercial gas separation membranes in comparable gas industries are discussed in terms of their potential in helium separation. Also presented are the various membrane process designs patented for the recovery and purification of helium from various sources, as these demonstrate that it is viable to separate helium through currently available polymeric membranes. This review places a particular focus on those processes where membranes are combined in series with another separation technology, commonly pressure swing adsorption. These combined processes have the most potential for membranes to produce a high purity helium product. The review demonstrates that membrane gas separation is technically feasible for helium recovery and purification, though membranes are currently only applied in niche applications focused on reusing helium rather than separation from natural sources.

## 1. Introduction

Helium is a noble gas that has a wide range of applications in important scientific, medical and industrial applications [1,2]. These industries take advantage of helium’s very low boiling temperature and chemically inert nature [3]. Helium’s largest usage is as a coolant in magnetic resonance imaging (MRIs) in hospitals, as well as an inert gas in welding, a carrier gas in analytical and scientific equipment, helium/oxygen mixtures for deep-sea SCUBA divers and in pressurizing and purging of pressure vessels, such as in rocket technology. Only ~8% of the global helium market is for party balloons [1].

The global demand for helium is growing, driven mainly by demand in Asia, especially in the developing economics of China and India [1]. The global usage of helium has grown by 990 million cubic feet from 2000 to 2015, and as such the helium price has risen from US$50 to US$104 per thousand cubic feet in that time [4,5]. To meet this demand new helium production facilities have recently been commissioned in Qatar and Australia [6]. However, global production is anticipated to fall short of global demand over the coming decades [2]. The increase in the helium price is expected to make low quality helium reserves attractive and drive further interest in helium recovery and recycling in existing industries. However, conventional technologies for helium recovery and purification rely on cryogenic liquefaction followed by pressure swing adsorption (PSA), both of which are energy intensive, especially as the helium concentration decreases in the feed [7]. As such, alternative technologies that can separate and purify helium will increasingly be investigated because of the economic advantage they might potentially have over the conventional approach. Membrane gas separation is one such technology that has significant potential in processing and purifying helium. This review discusses the sources of helium that are currently used, as well as potential recycling applications, which present niche opportunities for membranes. The current state of polymeric and inorganic membranes for helium separation is presented and analyzed. A major focus of the review is the discussion of membrane process strategies for helium recovery, including those presented in the patent literature, given the importance of the membrane process in gas separation economics [8]. Hence, this review aims to inform the membrane research community on the potential application of helium separation.

## 2. Helium Sources

Natural gas is the primary source of helium for commercial purposes, where it has accumulated over eons as the result of radioactive decay of uranium and thorium in the Earth’s interior [3]. The largest reserves of helium-rich natural gas fields exist in Western USA, where helium is commercially separated through cryogenic liquefaction and pressure swing adsorption [3]. The helium concentrations are generally <1%, but high quality fields in New Mexico and Alaska USA have helium compositions of 4.05 and 2.54% respectively [3]. Other helium-rich commercial natural gas fields exist in Qatar, Australia, Poland and Algeria, and potential fields exist in Russia, Iran, Italy, Tanzania and India, with a limited list of fields around the world provided in Table 1. The most important fields in the USA are the Hugoton-Panhandle field located across Texas, Oklahoma and Kansas as well as the LaBarge field in Wyoming [9]. Separation of helium from air has only been suggested under the most extreme situations, because the concentration is 5.2 ppm [1].

Recycling sources of helium are associated with leakages and waste gas from applications such as pressure tank testing [1]. These generally consist of helium with a mixture of air, with a much higher helium concentration than found in natural gas. The helium concentrations in these applications can be up to 99%, but is often diluted with air due to direct exposure to the environment. However, the quantity of gas to be treated is generally very small and present only in niche industries. For example, helium leakage from modern MRIs is very low and helium pressure tank testing is intermittent. Hence, in many of these industries the focus is on reducing helium wastage.

The separation of helium from natural gas using membranes has been discussed and demonstrated since membranes were first commercially proven as a technology [11]. However, to the best of the authors’ knowledge, no large scale membrane plant currently exists that separates and purifies helium from natural gas. In part this is because pressure swing adsorption (PSA) dominates the industry [6]. Similarly, the recycling and purification of helium from industrial processes, such as coolant leakage and waste gas from pressure vessels is technically possible through membrane separation. The advantage of membranes in recycling processes is clearly demonstrated by the range of commercial membrane processes currently available for helium recovery from localized sources [12,13,14].

## 3. Polymeric Membranes

Gas separation through non-porous polymeric membranes is dependent on concentration of gas within the polymeric matrix, which is influenced by favorable intermolecular interactions between the gas molecule and polymer chain. The gas permeability (P) through a range of polymeric membranes has been widely measured, with permeability described through the solution-diffusion model, and dependent on the diffusivity (D) and solubility (S) of the gas within the polymer [15]:

(1) P=D×S

For rubbery polymers the solubility of a gas corresponds to the Henry’s Law constant (k_D_) [15]:

(2) $$S=k_{D}$$

The Henry’s law constant can be determined from Flory-Huggins theory of mixing, as it is related to the volume fraction of the amorphous polymer (φ_p_), the partial molar volume of the gas (V_R_), the molar volume of an ideal gas at STP (V_S_ = 22,410 cm^3^/mol) and the Flory-Huggins interaction parameter between gas and polymer (χ). Hence, the Henry’s law constant (k_D_) can be expressed as [16]:

(3) $$k_{D}=\frac{\phi_{p}V_{S}}{V_{R}p_{0}}\exp\left[-\left(1+\chi \right)\right]$$

For glassy polymers, the free volume between polymeric chains can accommodate additional gas sorption, and hence the solubility to that region is described by the dual-sorption theory. This is modelled through a Langmuir isotherm, dependent on the Langmuir affinity constant (b) and maximum capacity (C’_p_) of the free volume. Hence, the solubility for glassy polymers is modelled by [15]:

(4) $$S=k_{D}+\frac{{C\prime }_{H}b}{1+b}$$

For helium permeability, the inert nature of the gas means that the solubility within polymeric membranes will be significantly lower compared to other gases. The only interaction between helium and the polymer is through dispersion forces, and as such the dual-sorption model cannot be applied. For the Henry’s law region it can be argued that the Flory-Huggins interaction parameter (χ) is zero, as there will be no intermolecular interaction between helium and the polymer chain. Hence, the Henry’s Law constant of helium in any polymeric membrane will merely be a function of the volume fraction of the polymer. This argument is used in the sorption measurements of other gases in polymers undertaken by gravimetric analysis, where helium is used as a reference gas to correct for buoyancy [17]. Kamiya et al. [18] report χ values for helium in a range of rubbery polymeric membranes. These values are significantly higher than those reported for other simple gases, such as H_2_, N_2_, and CH_4_, because of the need to ensure the calculated Henry’s law constants is very low (Equation (3)). Kamiya et al. state that there is considerable error in their calculations because of the hypothetical value they assumed for the vapor pressure of the liquefied helium at the measurement temperature (p_0_). For glassy polymeric membranes, a similar argument can be made for helium solubility associated with the free volume region; in that the Langmuir affinity constant (b) will be zero and there will be no preferential sorption of helium in the free volume. Hence, the amount of helium within the polymeric membrane would be equal to that of helium in the gas surrounding the material, and the excess sorbed amount is zero [19]. Rather, helium sorption within the free volume region will be based on size exclusion alone. Hence, helium permeability is dependent on diffusivity through the polymeric membrane, rather than solubility.

**Figure 1:**
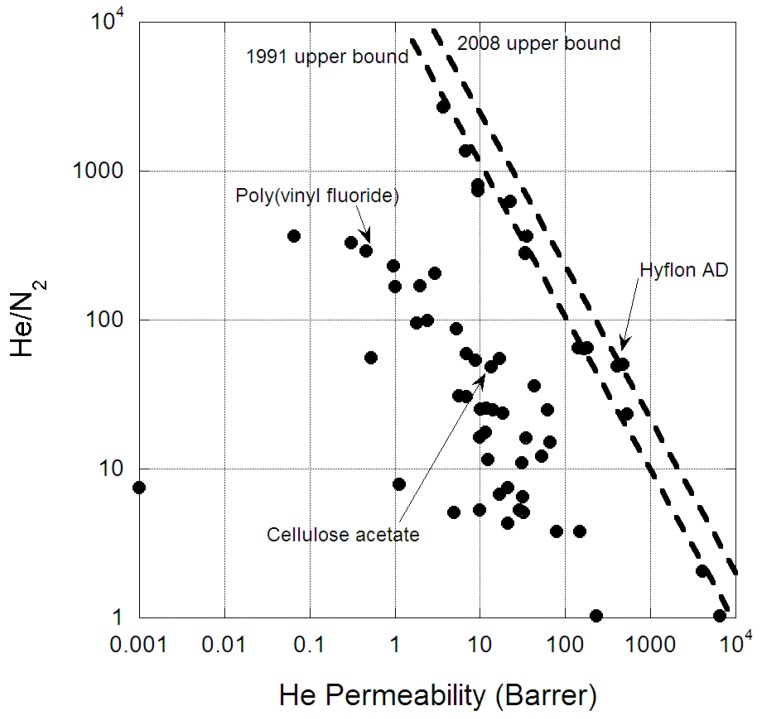
Permeability (Barrer) versus selectivity for polymeric membranes separating helium from nitrogen.

The helium permeability in a list of polymeric membranes is provided in Table 2. The Robeson’s plot of He against N_2_ is provided in Figure 1 and this has also been well reported in the literature [20,21]. All reported membranes have selectivity for He over N_2_, in part because of the smaller kinetic diameter of He compared to N_2_, ensuring He has a higher diffusivity through polymeric membranes [22]. Similarly, all glassy polymeric membranes have selectivity for He over CH_4_, however some rubbery polymeric membranes have selectivity for CH_4_. This is because of the strong solubility of CH_4_ in silicone-based polymers which dominates permeability through this class of polymer [23]. For both gas pairs, the highest permeable polymers are poly (trimethylsilylpropyne) (PTMSP) and substituted polyacetylenes, which are well known to have high fractional free volumes, which are almost microporous in structure [24,25]. Alternatively, high selectivity for both gas pairs is achieved for dense polymers such as polypyrrolone. For helium there is the standard trade-off between permeability and selectivity, with the upper bound clearly present for He/N_2_ separation (Figure 1). The upper bound for He/N_2_ gas pair has only improved slightly since Robeson first reported the behavior [20,21]; this may be an indication of the little research interest in helium separation from nitrogen. Furthermore, the gradient (λ) of these upper bounds corresponds well to established theory [26], based on the ratio of kinetic diameters (d):

(5) $$\lambda_{A/B}={\left(d_{B}/d_{A}\right)}^{2}-1$$

To the best of the authors’ knowledge no polymeric membrane has been successfully commercialized for He recovery and purification from natural gas directly or as part of a natural gas processing plant. However, a range of commercial membranes exist for acid gas removal which are used in natural gas processing, and these may have the capability for helium recovery. Asymmetric cellulose acetate is reported to have a He permeance of 106 GPU, a He/N_2_ selectivity of 34 and a He/CH_4_ selectivity of 31 [27], which places it in the middle of the Robeson plots. Hence, cellulose acetate-based commercial Cynara (Cameron) and Separex (Honeywell UOP) membranes, currently used in the removal of H_2_S and CO_2_ from natural gas, may be viable in helium recovery applications. Cellulose acetate membranes have been used commercially for hydrogen recovery [28], which can be comparable to helium recovery given the similarity in size of these two molecules. Indeed, other commercial polymeric gas separation membranes, such as Prism (Air Products), Medal (Air Liquide) and Ube Industries, have been commercialized for hydrogen separation [28] and hence there is opportunity in helium recovery. It is expected that polymeric membranes for helium separation will be an active research and commercialization area in the future. In particular, research is expected to focus on improvements in He/N_2_ and He/CH_4_ selectivity, as the low concentration in natural gas favors higher selectivity membranes to achieve the desired recovery and purification [29]. Critically, helium solubility within polymeric membranes cannot be altered and so improvements to the selectivity are better achieved through changing the respective diffusivity. This would favor polymeric membrane materials that are denser and have lower fractional free volume, because this morphology would impact the diffusivity of the smaller He atom to a lesser degree than the diffusivity of N_2_ and CH_4_.

## 4. Inorganic Membranes

Inorganic membranes have also been investigated for helium separation; the performance of a number of them is provided in Table 3. Inorganic membranes have a number of advantages over polymeric membranes, for example their ability to withstand harsher conditions such as high temperatures and corrosive gases [43]. However, the focus in the literature is on hydrogen separation, with helium permeation being one of the gases used in characterization rather than as a specific application. All of the inorganic membranes presented here are porous and hence it is difficult to achieve the selectivities observed for polymeric membranes (Table 2). The inert nature of helium means that surface diffusion and capillary condensation will not occur for porous membranes. However, many of the reported inorganic membranes have selectivities above the Knudsen diffusion selectivity (He/N_2_ is 1.9, He/CH_4_ is 2) [44] indicating that molecular sieving is occurring, where the kinetic diameter of He is 2.6 Å, N_2_ is 3.64 Å and CH_4_ is 3.8 Å [45].

Metal organic framework (MOF) membranes provide high selectivities for inorganic-based membranes, in part because the framework morphology can be specially designed to enable helium diffusivity while hindering other gases permeation. Similarly, microporous silica prepared by silica sol deposition on alumina support layer membranes achieve very high helium permeances and selectivities [53,54]. This is because the resulting silica pores’ diameters are of molecular size and hence excellent molecular sieving is achieved, while the deposition technique is able to fabricate a consistent layer without any defects. Thus, there is potential for microporous silica membranes to also produce high purity helium product from many of the aforementioned sources, however fabrication on large scales remains an issue. To the best of the authors’ knowledge no commercial inorganic membrane module exists for helium separation. Inorganic membranes will be an active area of research into the future, however it is expected that helium separation will be considered a minor application.

## 5. Membrane Processes

For membranes, both polymeric and inorganic, to be viable for helium recovery, the process design needs to be economically competitive against conventional technology. Therefore a major focus of membrane research in helium recovery will be on developments to the process designs that achieve the aims of low energy duty and minimizing equipment sizing, including membrane area. In other gas separation applications, it has been demonstrated that improvements in process design have a stronger influence on membranes’ economic competitiveness than just increasing the permselectivity of the membrane [56], and this argument also holds for helium recovery.

**Figure 2:**
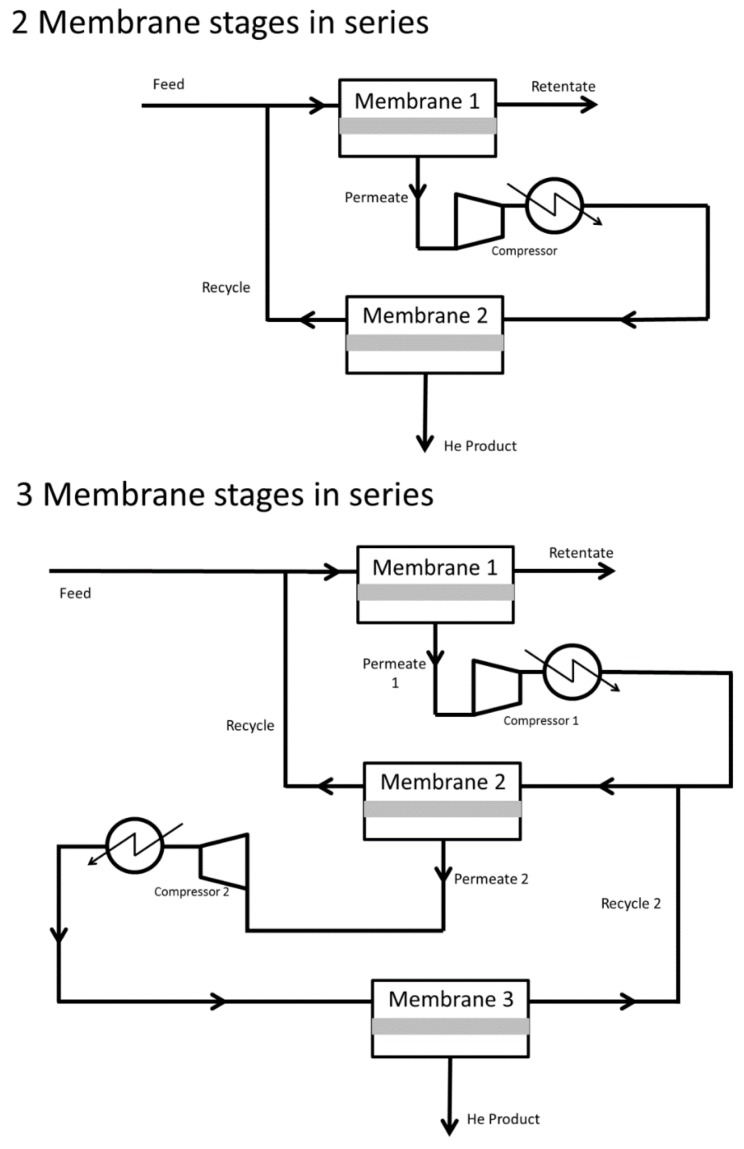
Process diagram example of two and three membrane stages processes, with recycle streams, recovering helium.

There has been a range of membrane processes designed for the recovery of helium from different sources, both in the research and patent literature. For the recovery and purification of helium directly from natural gas, membranes have demonstrated that this separation is possible when combined as two and three stages in series, with recycle streams (Figure 2) [29,57,58]. These designs can enable natural gas with 1 mol % helium and greater to be purified to a very high concentration, while utilizing existing polymeric membranes that have high He/CH_4_ selectivities. For example, the Alaska field (Table 1—2.54 mol %) can be purified to 99% through a three stage process with a membrane based on Teflon FEP (He/CH_4_ selectivity of 44) operating with pressures of 10 MPa, or with a poly vinylchloride-based membrane (He/CH_4_ selectivity of 71.4) when the pressure is 5 MPa. In contrast, a Hyflon AD-based membrane (He/CH_4_ selectivity of 157) will be able to process the Alaska field through a three stage process with membrane pressures of 625 kPa [29]. However, to achieve this high separation and purification the stage-cut of the second or third membrane stage is marginal and the required pressure driving force across the membranes is substantial. Hence, this requires significant compression energy and is the reason that direct recovery of helium from natural gas through membranes has not been commercialized.

Membranes inclusion in natural gas processing for helium recovery is a more viable option, and there are a range of different processes in the patent literature. Two and three membrane stages in series, with recycles, have been demonstrated to recover and purify helium from the exit gas off the nitrogen rejection unit (NRU) [29]. This is because the helium concentration is higher than in natural gas, and separation is mainly from nitrogen. This separation can be achieved with He/N_2_ selectivities above 20 and reasonable compression ratios. Commercial cellulose acetate-based membranes should be able to undertake this separation as well as commercial polyimide membranes [27]. As such, there is little need for the development of novel membranes in this application, and rather the focus should be on fabrication of low cost modules and ultra-thin layers to enhance flux. Indeed, processing NRU exit gas should be a more straightforward operation than acid gas removal from natural gas, because water and higher hydrocarbons have been removed from the gas, which are known to effect membrane performance in acid gas removal [59].

**Figure 3:**
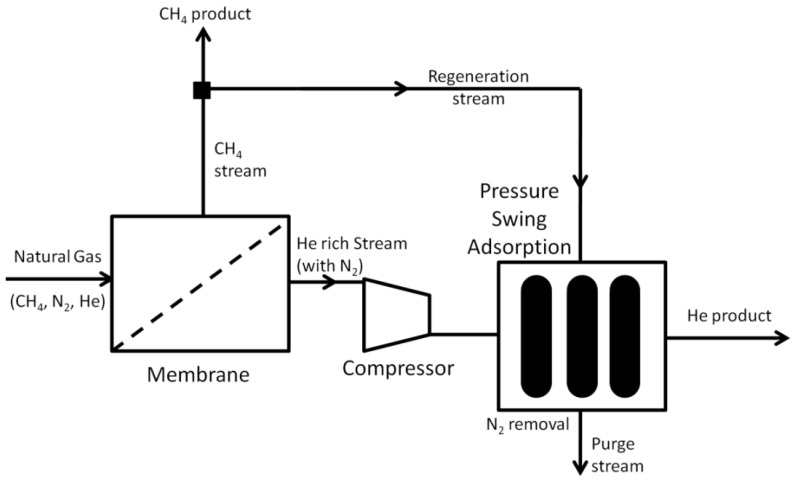
Process combining membrane separation with pressure swing adsorption (PSA) for the recovery and purification of helium.

The most viable membrane process for helium is the upgrading of crude helium to 90 mol % purity, which has energy duties comparable with other separation technologies. This requires a He/N_2_ selectivity of 10 for a 60 mol % feed, increasing to a He/N_2_ selectivity of 17 for a 50 mol % feed. This can be achieved with a variety of polymeric membranes (Table 2), including those membranes commercialized for natural gas sweetening. However, this process requires PSA to undertake the final purification stage and achieve ultra-high purity helium. Indeed, the majority of membrane designs processing helium consist of membranes used in series with another separation technology, generally PSA. This can consist of the membrane stage undertaking the first recovery stage and then sending the helium-rich stream to undergo final purification through another technology [60,61,62,63], as demonstrated in Figure 3. Or it can consist of another separation technology, such as PSA, undertaking the recovery of helium before the membrane stage achieves final purity specifications [64]. For PSA, the need to regenerate the adsorbent beds is critical, and a part of the novelty in the patent literature is to use the membrane’s retenate gas stream as the regeneration gas [65,66]. Additional novelty in many of these processes is the stream switching between the membrane unit and the PSA beds, and how they are interconnected to reduce compression requirements, recycle helium and regeneration of the adsorbent beds. Many of the patented systems of these hybrid membrane–PSA systems for natural gas are not specific for helium, but are rather broad in the gases discussed. This enables them to also cover hydrogen recovery from natural and refinery gas [67], which behaves very similar to helium recovery.

It is also possible to position membrane separation between two alternative separation technologies. United States Patent 5224350 describes such an invention [68], where a physical solvent absorption process is used to remove acidic gas from a natural gas stream. The off gas of this is rich in helium and nitrogen and therefore undergoes separation through a membrane unit to recover the helium. This helium product then undergoes PSA to produce the final purified helium product. It is therefore clear that standard membrane gas separation can be incorporated with a range of other separation technologies to achieve the required helium recovery and purification from natural gas. The process designs are achievable with polymeric membranes having reasonable He/N_2_ and He/CH_4_ selectivities, which are already present in commercialized membranes for natural gas sweetening. However, to the best of the authors’ knowledge no large scale implementation of these membrane processes has occurred for helium recovery from natural gas processing; though with continual interest in lower grade helium reserves there will be future interest in membrane process design developments in this area. It is expected that the continual improvements in membrane process design in other gas processing applications will also be applied to helium recovery. In particular, a focus on stronger integration with PSA is expected.

For other helium-rich applications, membranes are used to recover and recycle the helium product and there are a number of commercial units that can be purchased [69,70]. For example, Innovative Gas Systems (IGS) have their GENERON membrane for the recovery of helium from cryogenic, controlled atmosphere and electronic applications [13]. Their membrane process claims to produce a product purity of >90% and helium recovery >98%. Evonik Industries also markets their Sepuran membranes for the recovery and recycling of helium from a similar range of applications [12]. In atmosphere-helium mixtures, such as that required for deep sea diving, Divex Global markets their Helipure system, which uses a membrane to recover the helium from air mixtures and recompresses for recycling [14]. Their process claims a single membrane pass is able to reduce the nitrogen amount by a third and lower the oxygen amount to three quarters of their original concentration. Hence, there are clearly commercial opportunities for membranes in these niche industries and with the rising helium price it is anticipated that industry usages with recycle opportunities will also be commercially explored.

## 6. Conclusions

This review examines the current state of the literature for helium recovery and purification by membrane gas separation. The major developments in membranes technology will be in process developments, as currently available polymeric and inorganic membranes have the required permselectivity to achieve the desired separation in many of the potential helium recovery situations. Helium can be sourced from natural gas and related gas processing, as well as from the recycle and recovery associated with niche industrial applications. A wide range of polymeric membranes have been investigated for helium separation, with standard Robeson plot behavior observed against nitrogen and methane. Inorganic membranes have also been reported for helium separation, though their selectivities are lower than polymeric membranes because of their porous nature. A range of membrane process designs have been reported for recovery and purification of helium. Two and three stage membrane processes can recover helium directly from natural gas as well as from the nitrogen rejection unit exhaust gas, though they require considerable compression duties. The patent literature has a strong focus on combined processes where membranes are in a series with another separation technology to achieve both high helium recovery and high helium purity in the final product. This other separation technology is most often pressure swing adsorption, and the gas stream switching and connectivity between the PSA unit and membrane are of particular interest to reduce the energy duty of helium recovery and increase the viability of the process design.

## Figures and Tables

**Table 1:** List of helium-rich natural gas fields and their composition (mol %) [3,10].

Natural Gas Field	He	CH_4_	N_2_	CO_2_	C2+
New Mexico, USA	4.05	49	45	0.90	1.05
Alaska, USA	2.54	90.2	6.8	0.3	–
Texas, USA	1.17	66.2	31.1	0.10	1.43
Alberta, Canada	0.53	93	6	0.50	–
Ostrow, Poland	0.40	56	46	0.30	0.30
North Field, Qatar	0.03	79.5	5.19	3.68	8.85
Palm Valley, Australia	0.21	97.5	2.3	0.10	–

**Table 2:** He permeability (Barrer), He/N_2_ and He/CH_4_ selectivities in a range of polymeric membranes.

Polymer	He Permeability	He/N_2_	He/CH_4_	Ref.	Citations
Poly(trimethylsilylpropyne)	4100	2.05	0.98	[30]	5
Poly(trimethylsilylpropyne)	6500	1.03	0.433	[31]	74
Substituted Poly(diphenylacetylene)	11200	0.97	0.38	[25]	33
Substituted Poly(diphenylacetylene)	15800	1.01	0.46	[25]	33
Substituted Poly(diphenylacetylene)	12800	1.07	0.46	[25]	33
Substituted Poly(diphenylacetylene)	17800	1.07	0.51	[25]	33
Substituted Poly(diphenylacetylene)	13700	1.05	0.47	[25]	33
Isotactic poly(methyl methacrylate) (PMMA)	3.75	2679	–	[32]	51
Atactic PMMA	9.43	806	–	[32]	51
Syndiotactic PMMA	9.57	736	–	[32]	51
Poly(trichloromonochloroethylene) poly diacetylene (PDA)	34.1	284	–	[15]	1384
Nafion 117	40.9	–	401	[33]	93
Poly(trichloromonochloroethylene)	34.1	–	406	[15]	1384
Tetramethyl bis polycarbonate	206	–	43.8	[15]	1384
Poly(vinyl alcohol)	0.0071	–	–	[34]	67
Poly(vinyl alcohol)	0.052	–	–	[15]	1384
6FDA-DAF polyimide	98.5	–	156	[35]	73
6FDA/tetramethyl PDA polyimide	530	23.2	–	[15]	19
Polyimide (6FDA-6FpDA:DABA (2:1))	142	65	–	[36]	31
Polyimide	396	–	–	[15]	1384
Polypyrrolone (6FDA/PMDA (10/90)-TAB)	22.5	622	3041	[37]	55
Polypyrrolone (6FDA/PMDA (25/75)-TAB)	35.7	364	1594	[37]	55
Polypyrrolone (6FDA-TAB)	166	64.4	184	[37]	55
Polyarylate (TMHFBPA I/T)	182	64.8	–	[38]	6
Hyflon AD	405	48.8	167	[16]	1186
Hyflon AD60X	476	50.3	157	[39]	43
Teflon AF-2400	3600	–	6	[40]	153
Teflon FEP	62	25	44	[7]	70
Viton E60 fluoroelastomer	30.5	–	–	[41]	19
Viton fluoroelastomer	43.9	–	–	[41]	19
Cytop	170	–	–	[16]	1186
Fluorinated polynorbornene	185	–	–	[42]	20
Hostaflon perfluoroalkoxy alkane (PFA)	43.9	35.9	41.8	[43]	–
Poly(tetrafluoroethylene-co-ethylene)	5.63	30.9	–	[43]	–
Poly(trifluorochloroethylene-co-ethylene)	5.33	87.5	–	[43]	–
Polyvinyl fluoride	1.8	95	280	[7]	70
Poly(vinyl fluoride)	0.46	289	–	[43]	–
Low density polyethylene (LDPE)	4.92	5.06	1.68	[41]	552
High density polyethylene (HDPE)	1.14	7.8	2.97	[41]	552
Poly(ethylene-co-propylene)	31.9	6.49	–	[42]	14
Poly(ethylene-co-propylene)	29	5.31	–	[42]	14
Poly(ethylene-co-propylene)	21.3	4.32	–	[42]	14
Poly(propylene)	0.373	0.85	–	[43]	–
Trespaphan	14.1	25	–	[44]	–
Trespaphan	11.96	25.3	–	[44]	–
Trespaphan	10.25	25.2	–	[44]	–
Trespaphan	11.6	17.6	–	[44]	–
Poly(styrene)	18.64	23.73	–	[45]	17
Polystyrene	35	16	15	[7]	70
Poly(ethyl methacrylate)	6.9	30.5	–	[43]	–
Poly(vinyl acetate)	12.57	–	398	[46]	418
Poly(trifluorochloroethylene)	6.79	1360	–	[47]	–
Poly(vinyl alcohol)	0.001	7.5	–	[47]	–
Poly(vinyl benzoate)	8.88	53.79	–	[48]	95
Poly(vinyl chloride)	2	168.5	71.4	[43]	–
Saran	0.31	330	260	[21,47]	47
Poly(butadiene)	32.6	5.06	–	[42]	14
Poly(butadiene-co-acryonitrile)	16.9	6.7	–	[49]	213
Poly(butadiene-co-acryonitrile)	12.3	11.5	–	[49]	213
Poly(butadiene-co-acryonitrile)	9.85	16.3	–	[49]	213
Poly(oxydimethylsilylene)	233	1.03	–	[43]	–
Nylon 6	0.53	55.8	–	[47]	–
Cellulose acetate	13.6	48.6	–	[47]	–
Cellulose nitrate	6.9	59.5	–	[50]	16
Ethyl cellulose	53.4	12.1	–	[50]	16
Polyvinyl fluoride	0.97	231	–	[51]	16
Polyvinylidene chloride	0.066	366	–	[51]	16
Nylon 6	2.43	98.8	–	[51]	16
Mylar	1.002	167	170	[21,51]	16
Polyethylene terephthalate	2.967	206	–	[51]	16
Cellulose acetate	1990	–	11.8	[26]	13
Silicone rubber	356	–	0.34	[26]	13
Phenylene silicone rubber	150	3.8	0.75	[7]	70
Nitrile silicone rubber	79	3.8	0.79	[7]	70
Polycarbonate	67	15	19	[7]	70
Trithene B	34	280	400	[7]	70
Ethyl cellulose	31	11	4.9	[7]	70
Ethylene-vinyl acetate	21	7.5	1.9	[7]	70
Viton A	17	55	110	[7]	70
Polyvinyl chloride	14	–	7	[7]	70

**Table 3:** He permeance (GPU), He/N_2_ and He/CH_4_ selectivities in a range of inorganic membranes.

Material	He Permeance	He/N_2_	He/CH_4_	Ref.	Citations
Ni doped silica	3466	–	600 (300 °C)	[46]	31
Porous Alumina	86,190	–	–	[47]	–
Isoreticular Metal-Organic framework (IRMOF-3)	2986	2.5	1.6	[48]	44
IRMOF-3 and -6	2389	2.6	1.3	[48]	44
Metal-Organic framework (MMOF)	32.9	3.5		[49]	176
[Cu_2_(bza)_4_(pyz)]_n_	8.1	3.9	7.3	[50]	32
[Cu_2_(bza)_4_(pyz)]_n_	1.76	–	–	[50]	32
Cu-BTC	4181	2.6	2.07	[51]	19
Hydroxy sodalite	–	8.8	5	[52]	–
Vycor Glas	4.8 Barrer	7619	–	[11]	70
Microporous Silica	2933	31	147	[53]	84
Microporous Silica	6570	560	–	[54]	65
Microporous Silica	89.6	–	5000	[55]	44
